# The Cercal Sensilla of the Praying Mantis *Hierodula patellifera* and *Statilia maculata*: A New Partition Based on the Cerci Ultrastructure

**DOI:** 10.3390/insects16111093

**Published:** 2025-10-24

**Authors:** Yang Wang, Xiaoqun Ding, Huan Li, Yang Liu

**Affiliations:** 1 Shangluo Research Center of Chinese Medicinal Materials Integrated Pest Management, College of Biology Pharmacy and Food Engineering, Shangluo University, Shangluo 726000, China; wyang369@163.com; 2 Key Laboratory of Resource Biology and Biotechnology in Western China, College of Life Science, Northwest University, Taibai North Road 229, Xi’an 710069, China; xiaoqun_d@163.com (X.D.); 18409231210@163.com (H.L.); 3 Department of Entomology, University of Manitoba, Winnipeg, MB R3T 2N2, Canada

**Keywords:** praying mantis, cercal sensilla, ultrastructure, scanning electron microscopy

## Abstract

**Simple Summary:**

Given that praying mantises rely heavily on their senses to hunt, navigate, and reproduce, this study used scanning electron microscopy to examine the sensilla on the cerci of two species, *Hierodula patellifera* and *Statilia maculata*. We found that both species share the same four types of sensilla, but their size, number, and distribution differ between species and sexes. Based on the specific combination and density of sensilla along the cerci, we propose a novel division into four distinct zones (I–IV). This zoning reveals a clear functional gradient: the base of the cerci is specialized for detecting strong mechanical stimuli relevant to reproduction, the middle part serves as a multimodal hub for integrating sensory cues during mating and oviposition, and the tip is simplified for close-range contact with the substrate. This zonal organization reflects adaptive evolution of the cerci for reproductive behaviors, such as mate localization in males and oviposition site assessment in females. This study provides morphological evidence for the adaptive evolution of the mantid sensory system.

**Abstract:**

Cerci function as crucial sensory organs in insects, featuring a diverse array of sensilla on their surface, analogous to those found on antennae. Using scanning electron microscopy (SEM), we characterized the ultrastructure and distribution of cercal sensilla in *Hierodula patellifera* (*H. patellifera*) and *Statilia maculata* (*S. maculata*). Results show that the cerci of *H. patellifera* and *S. maculata* are highly similar, with main differences observed in the number of cercal articles and the length of cerci. The cerci of both species and sexes are composed of multiple cylindrical articles, and there is variation in the number of types of sensilla on their surface articles within sex and individuals. Females possess more cercal articles than males, and their cerci are generally longer than those of males. In both sexes of these praying mantises, four types of cercal sensilla were identified: sensilla filiformia (Sf), sensilla chaetica (Sc), sensilla campaniformia (Sca) and cuticular pore (CP), with sensilla chaetica further classified into two subtypes (ScI, ScII). Sc are widely distributed over the entire cerci, while Sf are distributed in a circular pattern on the cercal articles. While the overall distribution patterns of cercal sensilla were conserved between the sexes, significant sexual dimorphism was observed in the morphological parameters of the sensory hairs, including their quantity, length, and basal diameter. Based on distinct sensilla arrangements on the cerci, we propose a novel zoning of the cerci into four parts (I–IV), which reflects a functional gradient specialized for reproductive roles: the proximal region is enriched with robust mechanoreceptors likely involved in mating and oviposition, the central region serves as a multimodal hub for integrating courtship and mating cues, and the distal region is simplified for close-range substrate assessment. These findings highlight the adaptive evolution of cercal sensilla in relation to reproductive behaviors and provide a morphological basis for future studies on mantis phylogeny and sensory ecology.

## 1. Introduction

Insects depend on a diverse repertoire of sensilla to detect a wide range of environmental stimuli [1], including mechanical, chemical, and thermal cues. These sensilla are distributed across multiple body parts, such as antennae, palps (both mandibular and labial), and cerci [2,3,4]. They play an important role in maintaining the survival, reproduction and behavior regulation of insects. The sensilla of insects show significant species and morphological diversity among different species, and even show some differences between different sexes of the same insect [5]. Moreover, the types, morphology, distribution, quantity and function of sensilla are not the same in different organs [6]. This morphological and functional variation reflects niche adaptation and has made the typology, distribution, and sexual dimorphism of sensilla a focal research area in entomology.

There are various types of sensilla, each serving different functions. Current classification systems for insect sensilla are primarily based on three criteria. They are classified based on the presence or absence of pores and the structure of the pores, such as non-porous, apically pored, and microporous structures, on side walls [7]. They can also be classified based on physiological function as mechanoreceptors, chemoreceptors, thermoreceptors, and hygroreceptors [8]. Additionally, they can be classified based on morphological characteristics, resulting in nine types and numerous subtypes [9,10].

The cerci, a pair of whisker-like appendages located at the most posterior abdominal article, serve antenna-like sensory functions in most insects. Sensilla covered on the surface of the insect’s cerci can detect airflow, vibration and chemical signals, and participate in insect predation avoidance, courtship and attitude control [11,12]. In addition, cercal sensilla help insects sense some physical or chemical stimuli from inside and outside the body, thereby regulating and controlling their own physiological and behavioral responses, and play an important role in life activities such as foraging, mating and oviposition of insects. At present, the cercal sensilla that have been reported are sensilla trichodea, sensilla chaetica, sensilla rod-like, sensilla campaniformia, and sensilla filiformia [13,14]. Insects mostly rely on their antennae for olfactory and tactile input. Therefore, scholars at home and abroad mainly study the dense sensilla on the surface of insect antennae, while the research on cerci is very limited.

As a typical predatory insect, the praying mantis holds significant value in biological control and bionics [15]. Unlike the passive trap-based strategy of spiders [16], praying mantises are specialists that depend critically on acute sensory perception for prey detection and mate localization [17]. This obligate reliance on sensory input is underpinned by the absence of effective alternative strategies for prey capture, thereby rendering their sensory apparatus a subject of significant scientific interest. As an important sensory organ of the praying mantis, the cercal sensilla has unique biological research value. The morphological characteristics of its cercal sensilla may be closely related to its predation strategy, still relatively lacking. Although the ultrastructure and neural mechanisms underlying cercal sensilla have been extensively characterized in several insect orders, such as *Orthoptera* and *Blattaria* [18,19], comparable studies within Mantodea—particularly regarding praying mantises—remain relatively scarce.

As an ancient insect group with an evolutionary history dating back to the Jurassic period [20], mantises exhibit remarkable functional diversification. Currently, approximately 2500 extant species of mantises have been described worldwide, classified into 439 genera, 60 subfamilies, and 29 families [21,22,23]. The *Hierodula patellifera* (Serville, 1839) (Mantodea: Mantidae) and *Statilia maculata* (Thunberg, 1784) (Mantodea: Mantidae) belong to genera within the Mantidae family commonly found in East Asia [15], primarily inhabiting tropical, subtropical, and temperate regions. The habitat of the praying mantis is complex and constantly changing. They require well-developed cerci and antennae to perceive the complex environment of the outside world. Both the *H. patellifera* and the *S. maculata* have slender cerci and dense distribution of surface sensilla, which are typical. Field observations indicate that *H. patellifera* and *S. maculata* inhabit distinct vertical strata: the former predominantly occupies the canopy of tall trees, while the latter is commonly found in low-lying weeds and shrubs. This marked divergence in their microhabitat structure—ranging from open, elevated branches to complex, cluttered ground vegetation—presents contrasting mechanical environments. These differing environmental contexts provide an ideal opportunity to investigate whether and how the ultrastructure of their cercal sensilla, the primary abdominal tactile organs, has diverged. This study therefore aims to characterize and compare the cercal sensilla of these two species to elucidate any morphological differentiation that may correspond to their distinct ecological niches.

The study of the cercal sensilla is a prerequisite for the exploration of some important insect behaviors. Schneider reviewed the morphology, classification and function of insect sensilla (especially antennal sensilla) for the first time [24]. Gnatzy & Schmidt used SEM and TEM to study the cerci of *Gryllus bimaculatus*, elucidating the fine structure of their sensilla [25]. Although the ultrastructure of sensilla has been characterized in many insect groups, detailed comparative studies on the cercal sensilla of Mantodea are still lacking [26,27,28,29]. Currently, few studies have examined the types of sensilla on the cerci of praying mantis [30,31,32,33]. The expression characteristics of the ultrastructure and its relationship with ecological adaptability are not clear; in particular, whether gender dimorphism affects the functional differentiation of sensilla remains to be elucidated. This limits the understanding of the perception mechanism and ecological adaptation of the praying mantis. We hypothesize that the sexual dimorphism in cercal sensilla is driven by their distinct reproductive roles. Specifically, the cerci of females may have undergone adaptations that assist in the precise assessment of oviposition substrates and the physical molding of the ootheca, whereas in males, cercal sensilla may be optimized for detecting tactile or vibrational cues during courtship and mounting, thereby increasing the efficiency and success of mating interactions. This study therefore undertakes a comparative SEM examination of the cerci in *H. patellifera* and *S. maculata*. We focus on characterizing the ultrastructure, distribution, and sexual dimorphism of cercal sensilla, with the goal of establishing a morphological basis for future research into the sensory ecology and systematics of Mantodea.

## 2. Materials and Methods

### 2.1. Insect Collection and Preparation

In this study, five male and five female specimens of *H. patellifera* and *S. maculata* were collected from Guangdong Province, China, during July–August 2024. Specimens were maintained in nylon cages (25 × 25 × 30 cm) at 25 ± 1 °C and 80 ± 5% RH, fed with *Drosophila melanogaster*, and provided with distilled water ad libitum.

Live adult insects were transported to the laboratory in a nylon mesh cage. Fruit flies were given as a food source, and the cage was equipped a water spray to maintain relatively humidity at high level. Sexes were distinguished by examining the genitalia under a dissecting microscope (SMZ745T, Nikon Precision Shanghai Co., Ltd., Shanghai, China).

### 2.2. Sample Preparation for Scanning Electron Microscopy (SEM)

Live adult insects were anesthetized with ethyl ether and then dissected to reduce tissue damage. The cerci were carefully cut under a stereomicroscope and immediately rinsed with phosphate buffer (pH 7.2). Cerci were rinsed three times with a phosphoric acid buffer (pH 7.2).

The specimens were rinsed with a KQ-250DM ultrasonic cleaner (KunShan Ultrasonic Instruments Co., Ltd., Kunshan, China) at 45 kHz for 3 min and then dehydrated using a graded ethanol series of 60%, 70%, 80%, 90% and 95% each for 10 min and followed by two washes in 100% ethanol (15 min each). The specimens were dried using an SCD-350M Critical Point Dryer (ShiAnJia Biotechnology Co., Ltd., Beijing, China) for 12 h. Dried samples were mounted on SEM stubs with double-sided copper adhesive tape, coated with gold in a GVC-1000 sputter coater (BeJing Gevee-tech Co., Ltd., Beijing, China) for 1 min, and viewed with an SEM3200 scanning electron microscope (CIQTEK Co., Ltd., Hefei, China) at a 7–10 kV acceleration voltage.

### 2.3. Data Analysis

Cercal sensilla were identified and categorized according to established nomenclature from Carle et al. [31] and Faucheux [33]. The cercal sensilla were observed and recorded with SEM, and their type, quantity, distribution, and length were clarified using these records. Morphometric parameters (length and diameter) were quantified using the SEM system’s integrated measurement tools. Image adjustments for optimal clarity were performed with Adobe Photoshop CC 2020 (Version 21.0.1, Adobe Inc., San Jose, CA, USA), and schematic distribution maps were generated using Adobe Illustrator 2025 (Version 29.8.1, Adobe Inc., San Jose, CA, USA). Diagrams were drawn using Origin Pro 2021 (Version 9.8.0.200, OriginLab Corporation, Northampton, MA, USA). All statistical analyses, including ANOVA followed by Tukey’s test (α = 0.05), were conducted with SPSS Statistics (Version 26.0, IBM Corp., Armonk, NY, USA).

## 3. Results

### 3.1. Gross Morphology

**Figure 1:**
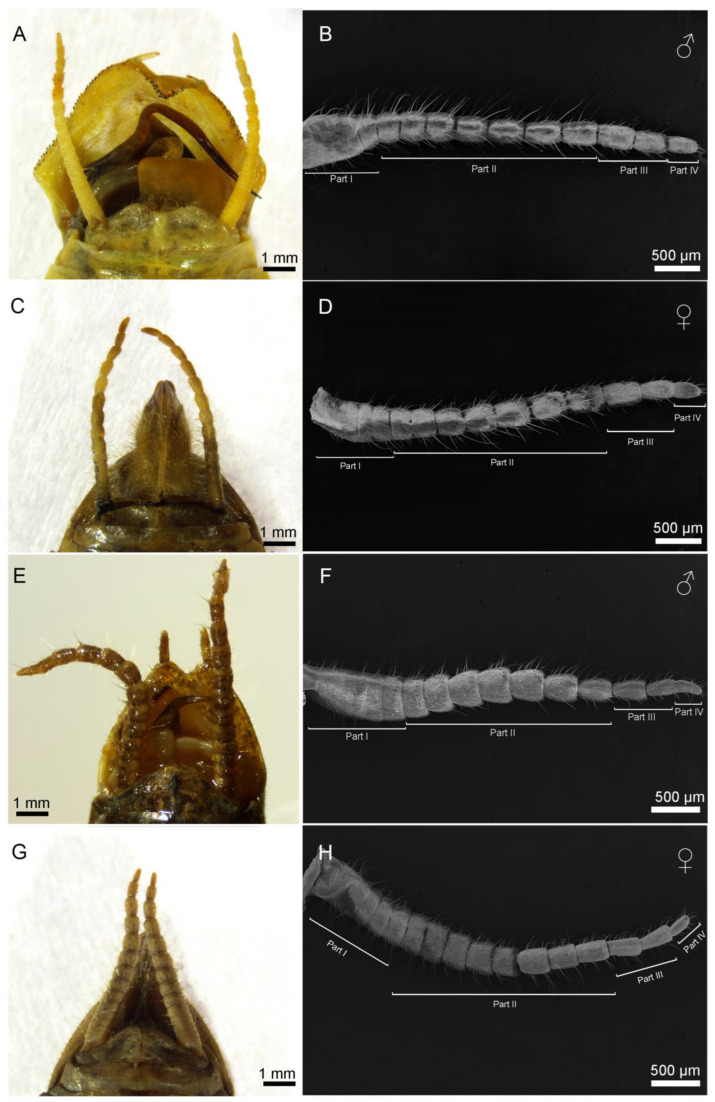
The dorsal view of gross morphology of male and female abdomen terminal and cerci. (**A**) The male genitalia capsule of *H. patellifera* showing the insertion of the cerci and their relative position; (**B**) The dorsal view of male cerci of *H. patellifera*; (**C**) The female ovipositor of *H. patellifera* showing the insertion of the cerci and their relative position; (**D**) The dorsal view of cerci of female *H. patellifera*; (**E**) The male genitalia capsule of *S. maculata* showing the insertion of the cerci and their relative position; (**F**) The dorsal view of cerci of male *S. maculata*; (**G**) The female ovipositor of *S. maculata* showing the insertion of the cerci and their relative position; (**H**) The dorsal view of cerci of female *S. maculata*. The scales are represented in each micrograph.

**Figure 2:**
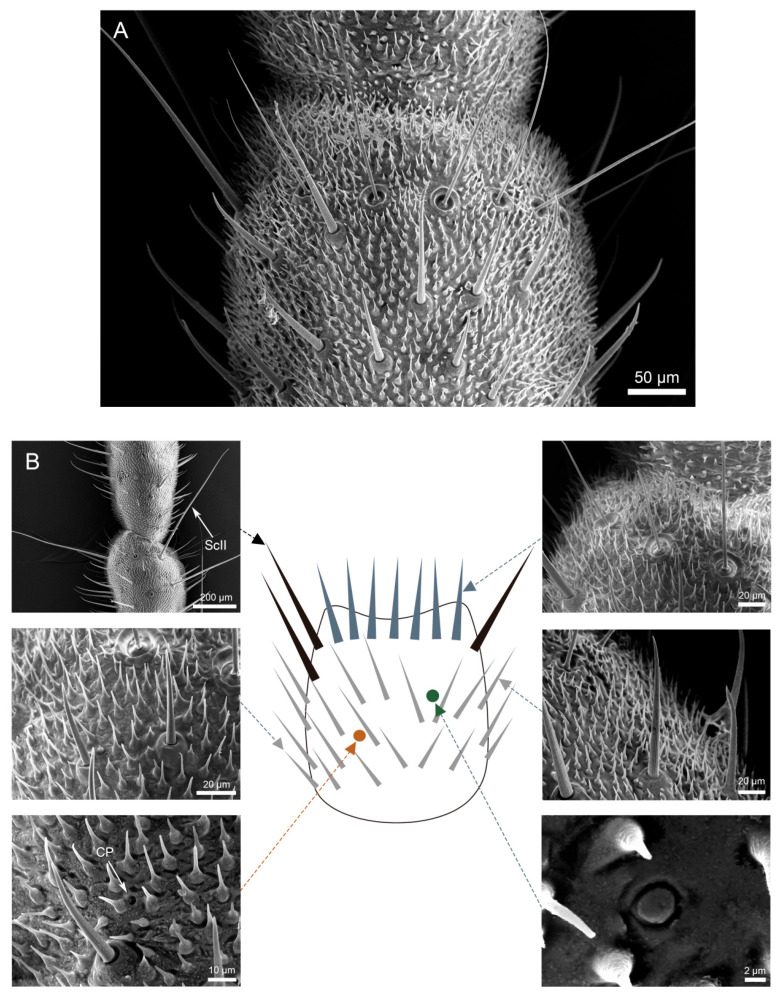
Scanning electron micrographs of cercal sensilla (**A**) and its schematic representation (**B**) in *H. patellifera*. Four different types of sensilla were identified: sensilla filiformia (Sf) (long blue triangle), sensilla campaniformia (green), cuticular pore (orange), and sensilla chaetica. Two different types of sensilla chaetica were distinguished depending on their localization and their length, and were termed ScI (gray) and ScII (long black triangle).

**Figure 3:**
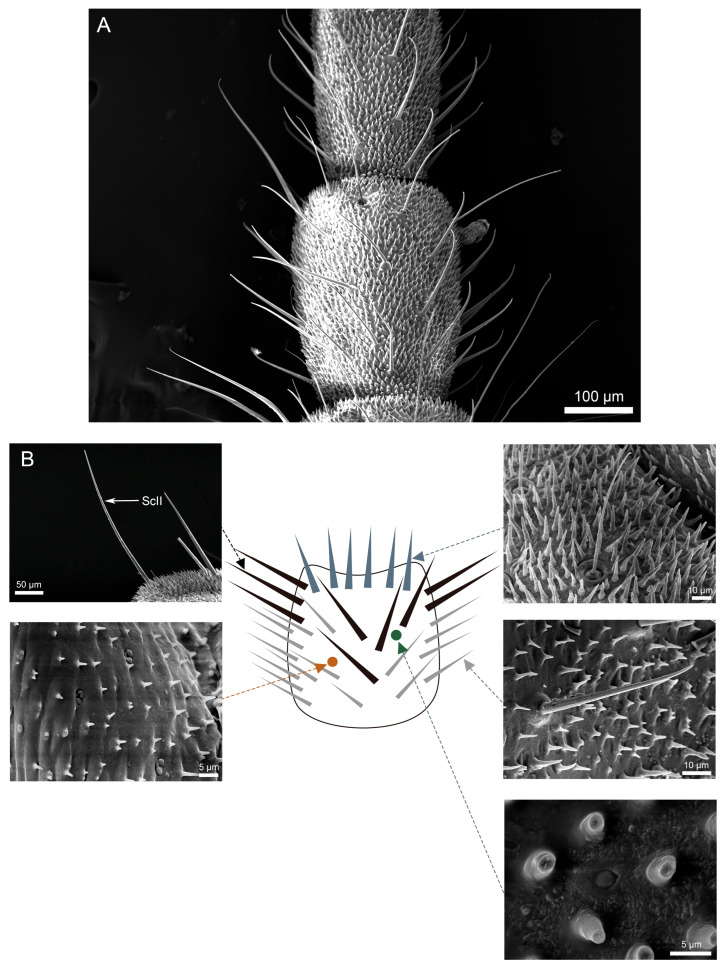
Scanning electron micrographs of cercal sensilla (**A**) and its schematic representation (**B**) in *S. maculata*. The schematic representation for *S. maculata* (Figure 3) follows the same conventions as described for *H. patellifera* (Figure 2).

The cerci of both *H. patellifera* and *S. maculata* exhibit a structure composed of multiple cylindrical articles that taper distally from the terminal abdominal article (Figure 1A,C,E,G). While overall morphology is similar between sexes, sexual dimorphism is evident in article number (Figure 1B,D,F,H): male cerci typically have 12–16 articles, with a predominant count of 14, whereas female cerci typically have 12–18 articles, with a predominant count of 16. Statistical analysis (independent samples *t*-test) confirmed that females possess significantly more cercal articles than males in both *H. patellifera* (t = −9.000, df = 8, *p* < 0.001) and *S. maculata* (t = −5.000, df = 8, *p* = 0.001). In *H. patellifera*, the total length of cerci is 5.74 ± 0.94 mm (mean ± SD, *n* = 5) in females and 3.96 ± 0.73 mm in males. In *S. maculata*, the total length of cerci is 4.87 ± 0.69 mm (mean ± SD, *n* = 5) in females and 3.89 ± 0.58 mm in males. Interspecific comparisons show *H. patellifera* cerci are longer than *S. maculata* in both sexes. The cercal surface was uniformly covered with short, shark tooth-like setae. While no sexual dimorphism was observed in their morphology, distinct interspecific differences were noted: *H. patellifera* exhibited slender, conical setae (Figure 2B), whereas *S. maculata* possessed shorter setae with scaly bases (Figure 3B).

### 3.2. Types and Characteristics of Sensilla

Scanning electron microscopy (SEM) analysis revealed dense coverage of sensilla and microtrichia across the entire cercal surface in both *H. patellifera* and *S. maculata* (Figure 1). Based on the morphology of external features of sensilla observed with the SEM, we classified the cercal sensilla into four types: sensilla filiformia (Sf), sensilla chaetica (Sc), sensilla campaniformia (Sca), and cuticular pore (CP) (Figure 2 and Figure 3). The Sc were further classified into two subtypes, based on distinct localization patterns and their length (ScI, ScII) (Figure 2B and Figure 3B).

#### 3.2.1. Sensilla Filiformia (Sf)

**Figure 4:**
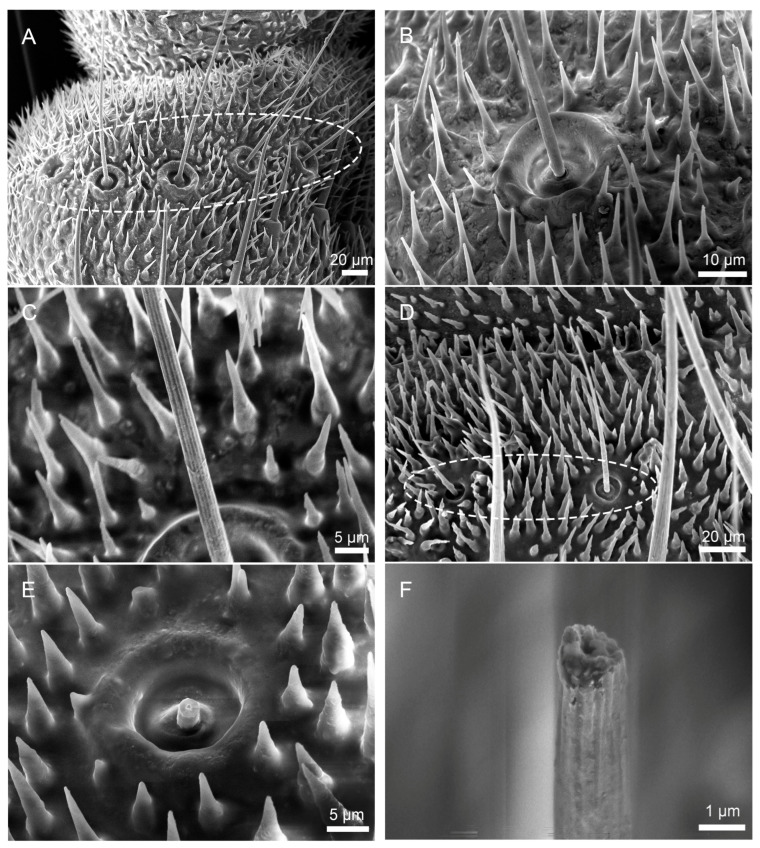
Morphology of Sf on cerci of *H. patellifera* and *S. maculata*. (**A**) Sf on the cerci of *H. patellifera*, showing overall morphology and monocyclic distribution of Sf; (**B**) High magnification image of the base of Sf of *H. patellifera*, showing hair fossa complex at the base; (**C**) The longitudinal grooves on the sensory hairs of *H. patellifera*; (**D**) Sf on the cerci of *S. maculata*, showing overall morphology and monocyclic distribution of Sf; (**E**) High magnification image of the base of Sf of *S. maculata*, showing hair fossa complex at the base; (**F**) *S. maculata* has longitudinal grooves and a hollow structure in cross-section.

Sf are distributed circularly near the distal margin of all articles excepting the terminal one (Figure 4A,D). The sensory hair is relatively curved as a whole. Sf is slender silky hairy, with a distinct hair follicle complex at the base (Figure 4B,E). This hair fossa complex is elevated on the surface of the cerci, and inside is a concave, subcircular contour resembling a caldera with a sunken center. There is a gap between the sensory hairs and the base, and a single sensory hair grows vertically from the nest, tapering from base to tip. Some indistinct longitudinal grooves are observed on the surface of these sensilla, extending from base to tip. (Figure 4C,F). In the cross-section of the sensilla filiformia (Sf) of *S. maculata*, a thick cuticle layer with a central pore is observed (Figure 4F). Further details regarding the cuticular structure and internal cavity characteristics require additional investigation via transmission electron microscopy (TEM).

For the female of *H. patellifera*, the average length and base diameter of Sf are 125.6 ± 49.3 μm and 23.0 ± 2.0 μm, respectively. For the male of *H. patellifera*, the average length and base diameter of Sf are 113.2 ± 50.3 μm and 18.1 ± 1.7 μm, respectively. The average length and base diameter for female *S. maculata* are 148.2 ± 66.0 μm and 22.2 ± 20.2 μm, respectively. For the male of *S. maculata*, the average length and base diameter are 155.2 ± 75.3 μm and 20.2 ± 2.8 μm, respectively. Statistical analysis revealed no significant sexual dimorphism in the diameter or length of Sf in either species (Table 1). Similarly, no significant interspecific differences in Sf diameter or length were found between the two species. However, it was observed that males of both species possessed a greater number of Sf on their cerci compared to females.

#### 3.2.2. Sensilla Chaetica (Sc)

**Figure 5:**
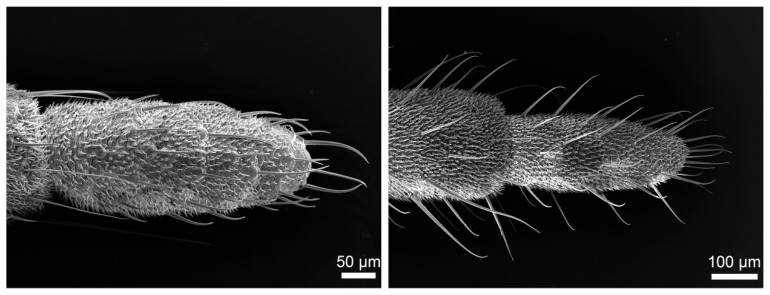
Comparative SEM illustrating interspecific differences in the angle of ScI between *H. patellifera* and *S. maculata*.

Sensilla chaetica (Sc) were the most abundant type of sensilla on the cerci of both *H. patellifera* and *S. maculata*. The angle of the ScI relative to the cercal surface differed between *H. patellifera* and *S. maculata*. In *H. patellifera*, the ScI was positioned at a very small angle, nearly flush against the cercal surface. In contrast, in *S. maculata*, the ScI was elevated at an angle of 60° to 90° (Figure 5).

**Figure 6:**
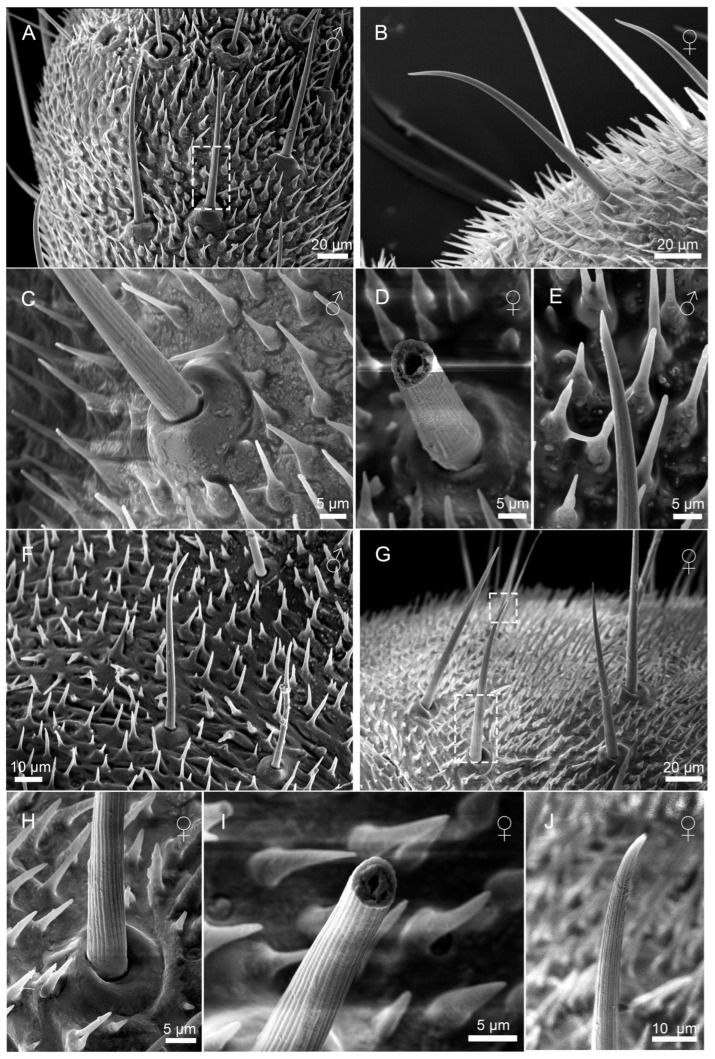
Morphology of (sensilla chaetica) ScI on cerci of *H. patellifera* and *S. maculata*. (**A**–**E**) Morphology of *H. patellifera*: (**A**) Overall morphology of ScI, *H. patellifera* male; (**B**) Overall morphology of ScI, *H. patellifera* female; (**C**) High magnification image of the base of ScI of *H. patellifera*; (**D**) ScI Sensory hairs truncated, showing hollow structure of *H. patellifera*; (**E**) High magnification image of the ScI tip of *H. patellifera* dotted line in (**A**). (**F**–**J**) Morphology of *S. maculata*; (**F**) Overall morphology of ScI, *S. maculata* male; (**G**) Overall morphology of ScI, *S. maculata* female; (**H**) High magnification image of the base of ScI of *S. maculata*, showing the deep grooves on the sensory hairs; (**I**) ScI Sensory hairs truncated, showing hollow structure; (**J**) High magnification image of the ScI tip of *S. maculata*, dotted line in (**G**).

Based on hair length, basal diameter, and distribution patterns, Sc were classified into two primary subtypes: ScI and ScII. ScI relatively straight throughout, stout at base. Slightly curved into an arc about two-thirds of the way from the base (Figure 6A,B,F,G). ScI basally inserted in movable basal fossa with upwardly elevated margins and a slightly concave center, with the margins of the basal fossa protruding conspicuously. (Figure 6C,H). Sensory hairs are more pointed apically (Figure 6E), with micropore (Figure 6J). The fracture surface of the sensory hairs of ScI shows that the inner part of the sensory hairs is a hollow structure with a thick cuticle layer (Figure 6D,I).

**Figure 7:**
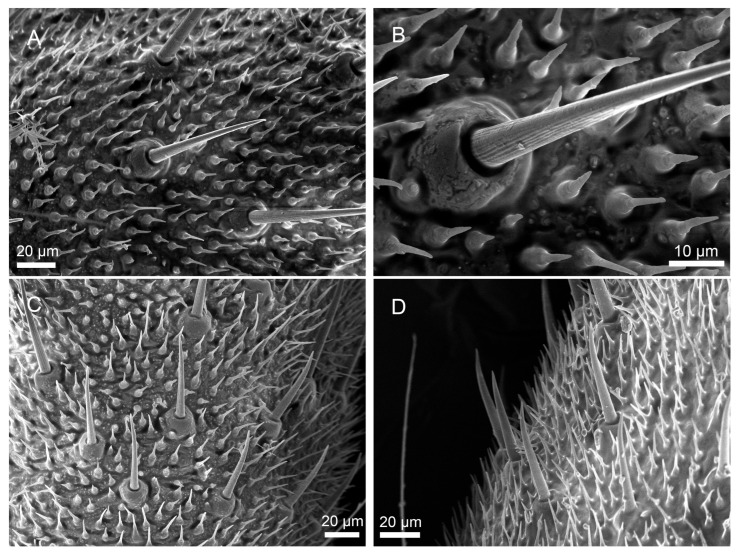
Morphological gradient variation of sensilla chaetica I (ScI) in *H. patellifera*. (**A**) Unique short, stout ScI overall morphotype. (**B**) High magnification image of the base of short, stout ScI; (**C**,**D**) Clustered distribution in the proximal to middle region and margins, absent in *S. maculata*.

**Figure 8:**
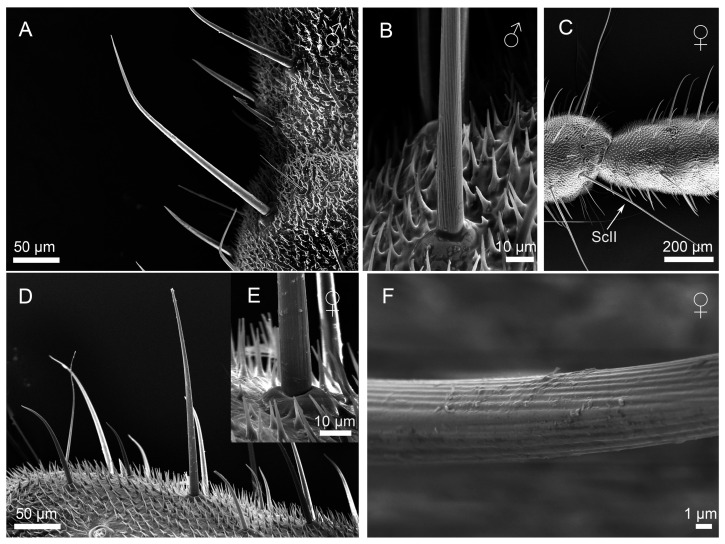
Morphology of sensilla chaetica II (ScII) on cerci of *H. patellifera* and *S. maculata*. (**A**–**C**) Morphology of *H. patellifera*. (**A**) overall morphology of ScII, *H. patellifera* male; (**B**) High magnification image of the base of ScII, showing the shallow grooves on the sensory hairs of *H. patellifera*; (**C**) Distribution of ScII in *H. patellifera*. (**D**–**F**) Morphology of *S. maculata*. (**D**) overall morphology of ScII, *S. maculata* female. (**E**) High magnification image of the base of ScII; (**F**) Showing the shallow grooves on the sensory hairs of *S. maculata*.

Notably, *H. patellifera* exhibited a continuous morphological variation in ScI, ranging from elongated forms to a unique short, stout morphotype (Figure 7). The base is ring-shaped with a noticeable protrusion, and there are obvious longitudinal stripes on the hair shaft extending from the base to the tip.(Figure 7B). The sensillar hair tapers from its base to a sharp point, curving downward near its tip. These stout ScI were clustered in the proximal-middle region of each cercal article (Figure 7C,D). In *S. maculata*, shorter Sc1 are only occasionally observed in some articles and are exceedingly scarce (Figure 8A). Given their rarity and sporadic occurrence, they are likely the result of individual variation and can be considered negligible in the general description of the species. In contrast, *S. maculata* generally exhibits only the elongated ScI morphology (Figure 6F,G), except in extremely rare instances.

Sexual dimorphism in ScI morphology was evident in both species. In *H. patellifera*, females had significantly longer ScI (78.2 ± 32.6 μm) compared to males (62.9 ± 20.4 μm; Table 1). However, there was no significant difference in diameter between males and females, which were 15.2 ± 2.4 μm and 17.8 ± 4.1 μm, respectively. Identically, *S. maculata* females displayed longer ScI (116.0± 22.9 μm) than males (94.4 ± 24.2 μm; Table 1). There was no significant difference in diameter between males and females, which were 16.766 ± 1.193 μm and 17.274 ± 3.101 μm, respectively. Interspecific comparisons revealed a significant difference in length, with values consistently greater in *S. maculata* than in *H. patellifera* within the same sex (*p* < 0.05), but not in diameter (Table 1).

ScII are the longest of all cercal sensilla (Figure 8A,D). The sensillar hair rise up vertically from the socket (Figure 8B,E), and the surfaces of ScII exhibit longitudinal grooves (Figure 8F). These sensilla are mainly concentrated on both sides of the base of each cerci near the end of the article (Figure 8C,D).

In *H. patellifera*, females exhibited significantly longer ScII (477.2 ± 76.0 μm) compared to males (274.0 ± 66.6 μm, *p* < 0.05), while basal diameters showed no significant intersexual difference (females: 24.5 ± 3.4 μm; males: 21.5 ± 3.0 μm). A similar pattern was observed in *S. maculata*, with female ScII measuring 250.5 ± 29.4 μm in length (vs. males: 220.3 ± 12.7 μm). But basal diameters females exhibited significantly thicker ScII (20.0 ± 3.3 μm) compared to males (17.0 ± 2.2 μm, *p* < 0.05). Statistically significant interspecific differences were observed between conspecific individuals of *H. patellifera* and *S. maculata* (Table 1). Overall, the diameter and length of ScII in *H. patellifera* were comprehensively larger than those in *S. maculata*, with the magnitude of interspecific differences in female individuals being greater than that in males.

#### 3.2.3. Sensilla Campaniformia (Sca)

**Figure 9:**
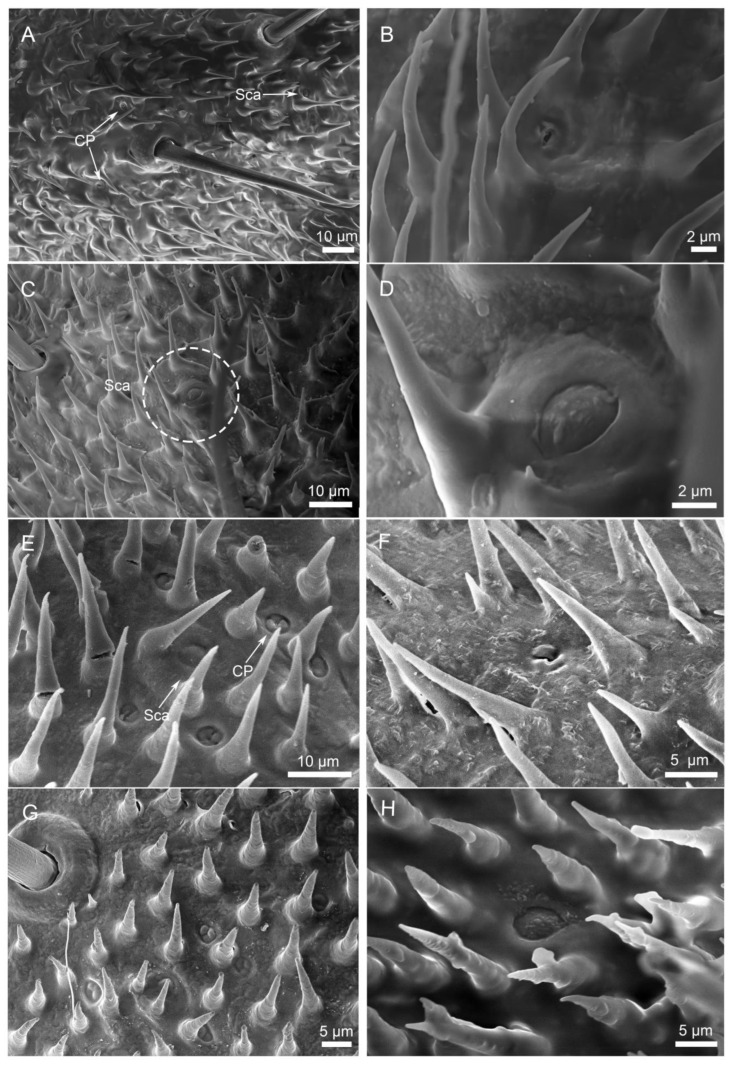
Morphology of Sca and CP on cerci of *H. patellifera* and *S. maculata*. (**A**–**D**) Morphology of *H. patellifera*. (**A**) The distribution position of Sca and CP in *H. patellifera*; (**B**) Morphology of CP of *H. patellifera*; (**C**) Distribution of sca near SC in *H. patellifera*. (**D**) High magnification image of Sca, dotted line in (**C**). (**E**–**H**) Morphology of *S. maculata*. (**E**) The distribution position of sca and cp in *S. maculata*; (**F**) Morphology of CP of *S. maculata*; (**G**) Morphology of CP at the base of the cerci; (**H**) Morphology of Sca in *S. maculata*.

Sca exhibit a dome-like structure with a pleated depression along the periphery, resembling a button (Figure 9A,E). These sensilla possess a smooth surface lacking a sensory hair (unlike other sensilla types), and are borne in a rounded basal fossa elevated on the surface of the cerci, which is well margined (Figure 9D,H). They were sparsely and irregularly distributed on each article, frequently adjacent to Sc (Figure 9C). No sexual or interspecific differences in diameter were observed (Table 1).

#### 3.2.4. Cuticular Pore (CP)

Numerous irregular cuticular pores (CPs) were observed on the surface of each cercal article in both *H. patellifera* and *S. maculata* (Figure 9B,E). These structures exhibited a slightly concave center containing micropores, but their morphology differed between species: in *H. patellifera*, CPs featured a distinct central pore (Figure 9B), whereas in *S. maculata*, the central region showed folded structures in addition to central pore (Figure 9F,G). CPs were predominantly concentrated in the middle articles of the cerci and consistently located near Sc or Sca with no sexual dimorphism. Some are single distribution, while others are aggregated distribution.

### 3.3. Density and Distribution Pattern of the Cercal Sensilla

**Figure 10:**
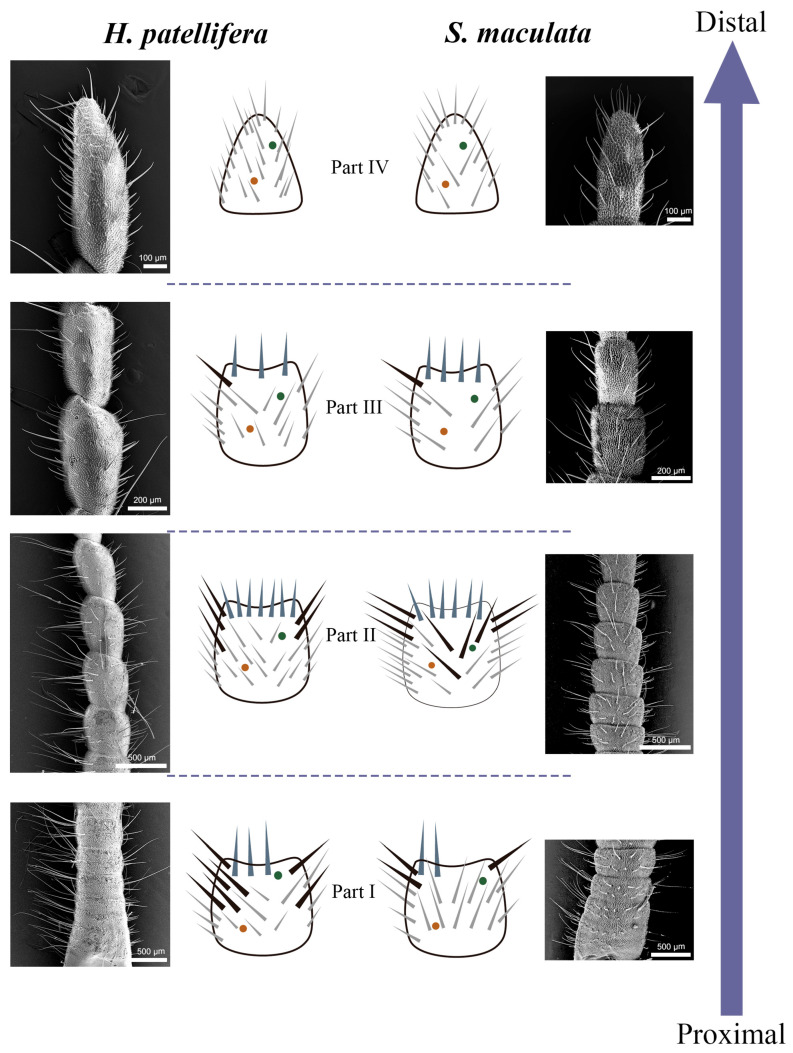
Schematic diagram of the distribution of sensilla on cerci. On the left is the distribution of cercal sensilla of *H. patellifera*, and on the right is the distribution of cercal sensilla of *S. maculata*. The schematic diagram of the cerci (Figure 10) follows the same conventions as described for the sensilla types in Figure 2 and Figure 3. (Note: All left-side views show the medial aspect of the cerci).

These four types of sensilla are not uniformly distributed along the cerci but are concentrated in specific regions, forming a clear morphological and functional gradient. Sf shows a circular distribution on the side near the end of the cerci except for the last section. (Figure 4A,D). Sc are the most numerous and widely distributed sensilla on the cerci. ScI are present in every article of cerci, while ScII was found on both sides of the middle and proximal articles of the cerci, usually more medial than lateral (Figure 6, Figure 7 and Figure 8). The number of Sca and CPs is lower compared to the Sc, and they are located near the Sc (Figure 9A,G). In *H. patellifera*, the ScI showed a unique gradient morphological change, which was not observed in *S. maculata*. These short and thick ScIs form a cluster distribution in the middle area near the base of each article of the cerci and on both sides of the cerci (Figure 7). In order to represent variations in the sensilla distribution pattern along the cerci’s longitudinal axis, we divided the article into 4 parts from proximal to distal (Figure 10). Each part is characterized as follows.

#### 3.3.1. Part I: Proximal Basic Perception Zone

In part I, from the proximal to article # 6/# 7, the boundary of articles is unclear, and the length of the articles are shorter than in other Parts (Approximately 1/2 of the length of Part II articles), exhibiting a compact structure. The diameter gradually becomes thinner (no shrinkage) with the transition from the base to Part II.

**Figure 11:**
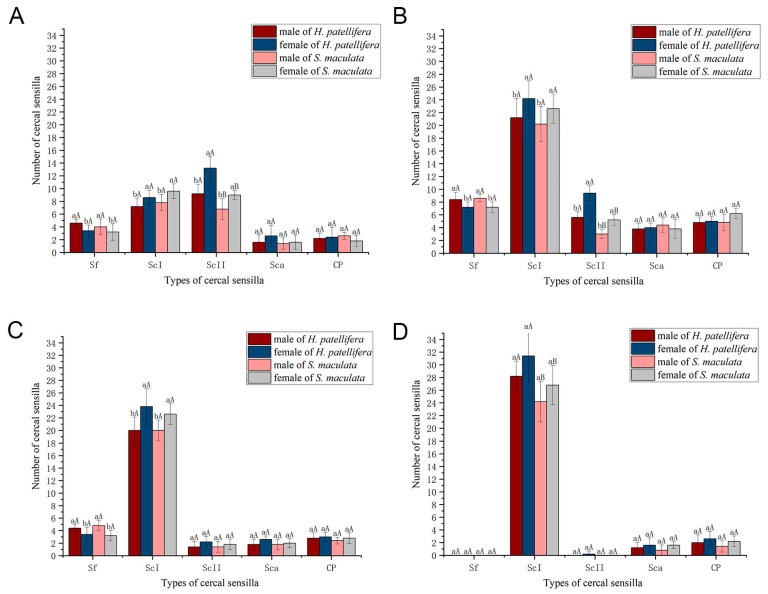
Distribution of sensilla on the cerci of *H. patellifera* and *S. maculata*. (**A**) Sensillar distributions on each article of cerci part I. (**B**) Sensillar distributions on part II of cerci. (**C**) Sensillar distributions on the article part III of cerci. (**D**) Sensillar distributions on part IV of cerci. Note: The quantitative analysis was performed based on the sensilla on the dorsal surface of the cerci. Error bars represent standard deviation (Mean ± SD, *n* = 5). Different lowercase letters indicate a significant difference between male and female within the same species (*p* < 0.05), and different uppercase letters indicate a significant difference between the same sex (*p* < 0.05).

In Part I of the cerci of *H. patellifera*, ScII are the most abundant sensillum type (Figure 11A). These sensilla are distributed on both the inner and outer surfaces of the cercal articles, with a significant difference in density between the two sides—specifically, the number of ScII on the inner surface is greater than that on the outer surface. In contrast, Part I of the cerci of *S. maculata* is dominated by two sensillum types: ScII and ScI. The distribution pattern of ScII in *S. maculata* is similar to that in *H. patellifera*: there is a significant density difference between the inner and outer surfaces of the articles, with more ScII present on the inner surface. Notably, ScI exhibit a consistent distribution pattern across both species—they are relatively uniformly distributed on the entire surface of the cerci and along the proximal sides of each cercal article. The difference is that there are a large number of short sc1 clusters distributed in each section or the proximal end of the cerci on both sides in *H. patellifera*.

**Figure 12:**
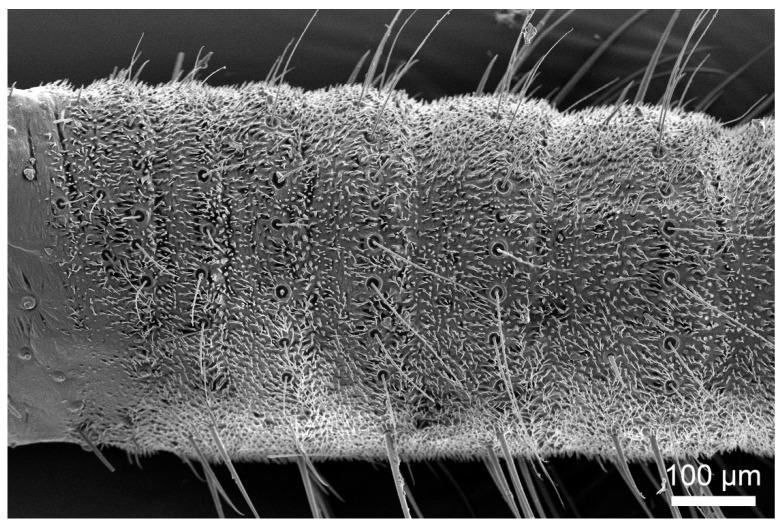
SEM showing ventral distribution of sensilla filiformia (Sf) on proximal cerci (Part I) of *H. patellifera*.

Sf are distributed in small numbers at the distal end of each article, and there are more Sfs in *H. patellifera* than in *S. maculata*. Generally, males possess slightly more Sf cells than females. Interestingly, in *H. patellifera*, the ventral surface of the proximal articles exhibited a distinctive feature: a high-density cluster of Sf (Figure 12). In contrast, the ventral surface of *S. maculata* showed a distribution pattern similar to its dorsal side, with no such specialization. The density of Sf in this ventral cluster was markedly higher compared to on the dorsal surface of the same articles and to any region on the cerci of *S. maculata*. Sca and CP showed a small amount of irregular sparse distribution around Sc in this part, and there was no significant gender difference and interspecific difference.

#### 3.3.2. Part II: Central Comprehensive Perception Zone

In part II (article # 7/# 8 to # 13/# 15), the article is relatively long, with clear boundaries and the highest sensilla density (Figure 11B). All types of sensilla are present, with a significant reduction in the number of ScII. The distribution pattern of ScII in *H. patellifera* is the same as in Part I, while in addition to their distribution on both sides of the cerci, there is also a small number of ScII in *S. maculata* are irregularly distributed on the middle surface of the cerci. ScI is similar to the first part distribution pattern and exhibits a consistent distribution pattern across both species. The Sf is circularly distributed at the distal end of each article of the cerci; the number is significantly higher than in Part I. A small amount of Sca and CP are scattered around Sc, and the number of Sca and CP is higher than in other regions.

#### 3.3.3. Part III: Distal Fine Perception Transition Zone

In part III (article # 14/# 16 to # 15/# 17), the articles gradually become thinner, and the sensilla density decreases compared to in Part II in both species (Figure 11C). The number of ScII decreased sharply or disappeared completely. ScI remains distributed but with slightly lower density, and its distribution pattern is similar to that in the second part, playing a dominant role. The short ScI clusters of *H. patellifera* have disappeared or decreased in number, and are now only found on both sides of the cerci. Sf persists but in reduced numbers, with greater variation in length. Sca and CP are extremely rare and are distributed only in the middle of the articles.

#### 3.3.4. Part IV: Terminal Specialized Perception Zone

In part IV (the most distal article), the cerci diameter is the thinnest, and the tip is pointed and rounded. The sensilla types in this section are the most simplified, with only ScI, Sca, and CP existing. The number of sensilla in this section is reduced, and ScI demonstrates its advantages (Figure 11D). The distribution pattern of ScI in *H. patellifera* is similar to that in Part III, evenly distributed on the surface and both sides of the body articles and the tips of the cerci. The terminal edge of the article body of *H. patellifera* has a relatively large distribution of short ScI. The ScI also shows a uniform distribution, and all are slender in *S. maculata*. Sca and CP are extremely rare in this region and are distributed in the central part.

## 4. Discussion

Insect sensilla exhibit a wide range of types [24], different insect have different sensilla, and different sensilla have different physiological functions. In this study, the cerci and sensilla of *H. patellifera* and *S. maculata* were studied for the first time by SEM. It is important to note that the functional interpretations presented herein are inferred from the external morphology, distribution, and well-established structure-function relationships of homologous sensilla in other insect groups, primarily *Orthoptera* [11,25]. Definitive confirmation of these proposed functions requires future electrophysiological studies.

Morphological observation revealed that the cerci of both *H. patellifera* and *S. maculata* maculata are composed of multiple cylindrical articles. This segmented structure likely enhances flexibility and facilitates multidirectional sensing. Compared to the antennae, the mantis cerci are more robust, a morphology likely associated with their direct involvement in reproductive processes such as mating and oviposition. Beyond their sensory role, the cerci appear to provide crucial physical support during these activities. For instance, in females, they may assist in shaping and stabilizing the newly formed ootheca. Furthermore, similar to *Tachycines lalinus* [34], the overall morphology of the cerci in both *H. patellifera* and *S. maculata* maculata shows no significant sexual dimorphism. However, a notable difference was found in the present study: in both mantis species, the cerci of females are longer and consist of more articles than those of males, whereas no significant difference in cercal length was observed between female and male *Tachycines lalinus*. We hypothesize that the elongated cerci in female mantises may be an adaptation to their unique reproductive requirements. In different mantis groups, the ootheca exhibits a species-specific morphology. Behavioral observations of oviposition indicate that the female’s cerci are involved in the processes of extruding, positioning, and molding the ootheca from the abdomen. During this process, females must precisely anchor the ootheca to a suitable substrate, a task potentially facilitated by the enhanced sensory or mechanical capabilities provided by the longer cerci.

Our scanning electron microscopy results indicate that the two mantis species share the same basic types of cercal sensilla, suggesting a degree of evolutionary conservation in sensilla typology. Inter-species differences are primarily reflected in the size, number, and distribution of sensory organs, which may be related to their microhabitats. *H. patellifera* inhabits the canopy of tall trees, whereas *S. maculata* is active in low-lying shrubs and weeds. This likely represents an adaptive evolution of their sensory systems to distinct ecological niches.

In *H. patellifera* and *S. maculata*, we observed four types of sensilla, including sensilla filiformia (Sf), sensilla chaetica (Sc), sensilla campaniformia (Sca) and cuticular pore (CP).

Sensilla filiformia (Sf), which possess a hair socket complex at their base from which elongated sensory hairs insert vertically, have been shown by electrophysiological studies to be particularly sensitive to air vibrations [19]; each Sf is generally composed of a single sensory cell and three enveloping cells and functions to detect airflow and mechanical vibrations [35]. For instance, in *Troglophilus neglectus*, whose cerci are perpendicular to the body, the Sf enable the detection of horizontal air currents [36]. In both mantis species studied here, the Sf are distributed nearly vertically along the interarticular boundaries, adjacent to the distal end of each article. This arrangement suggests an adaptation for maximizing contact with airflow. Therefore, the widespread distribution of Sf supports a potential role in detecting airflow changes, which could enhance sensitivity to environmental stimuli. In our study, the greater abundance of Sf sensilla observed in males of both mantis species may be functionally correlated with their active role in mating behavior. Males typically initiate mate searching and localization through more active flight and movement compared to females. They must also execute precise leaps onto the female’s back to achieve copulation and retreat promptly afterward to avoid sexual cannibalism. Throughout this behavioral sequence, rapid and accurate mechanosensory feedback is likely essential. We therefore hypothesize that the enriched Sf sensilla in males may improve mating success and survival by enhancing the detection of tactile and aerodynamic cues during critical mating behaviors.

Beyond detecting prey-generated air movements, the cercal Sf system may have an additional role in sound perception. Some mantis species are known to produce defensive sounds when threatened [37], and the cerci could potentially contribute to a multimodal auditory system alongside a specialized metathoracic auditory organ [38]. Research on crickets has established that filiform sensilla are tuned to specific frequency bands based on their length and mechanical properties [39,40]. The considerable variation in Sf length observed in both *H. patellifera* and *S. maculata* (Table 1) suggests the presence of a sensilla array that could be sensitive to a broad frequency range. This morphological feature might therefore allow the cerci to detect not only low-frequency air currents but also higher-frequency components of conspecific or defensive sounds.

Sc are generally known to be mechanoreceptive [11]. In the antennae of the camel cricket (*Tachycines asynamorus*), Sc are also located around other sensilla, serving a protective role [41]. In *Gryllus bimaculatus*, Schmidt & Gnatzy (1972) identified three types of bristles (sensilla chaetica) on the cerci: the longest type functioned as pure mechanoreceptors, while two shorter types possessed apical pores and were innervated by different numbers of sensory cells, suggesting a combined mechano- and chemosensory function [25]. On the cerci of both mantis species studied, Sc were the most abundant sensilla and were classified into two subtypes (ScI and ScII) based on their morphology and distribution. The widely distributed ScI are characterized by their relatively short length and the possession of a single apical pore (Figure 6J), the latter being a universal feature consistent with findings in orthopterans like *Gryllus bimaculatus* [25]. They are therefore likely capable of sensing mechanical stimuli as well as detecting chemical cues upon contact with surfaces or other organisms [42]. The unique short, stout morphological variant of ScI, found clustered in the proximal-middle region of articles *in H. patellifera* (and largely absent in *S. maculata*), likely represents an adaptation for perceiving stronger mechanical stimuli.

ScII is the longest sensory hair on the cerci, distributed on both sides of the cerci near its proximal end. Their length and distribution might cause them to respond first to external mechanical stimuli. Thus, they could play a protective role for other sensilla [43], and based on their morphological similarity to the long, purely mechanosensitive bristles in *Gryllus bimaculatus* [25]. ScII exhibited the most pronounced sexual dimorphism, being significantly longer and/or thicker in females. Considering the proximal location of the cerci near the abdominal terminus and genitalia, we propose that the well-developed ScII in females may play a crucial role in oviposition behavior. This functional disparity may be ecologically adaptive: female mantises, which are typically larger and bear the energetic costs of oogenesis and oviposition, may rely on enhanced mechanosensory acuity for selecting suitable oviposition sites, detecting threats while in a vulnerable Ootheca-laying posture, or sensing subtle vibrations during courtship and mating interactions.

Sca are very small and few in number. This type of sensillum has been found in many species of insects which belong to Hymenoptera, Hemiptera, Coleoptera, and Diptera [44,45]. Some studies suggest that Sca function as mechanoreceptors [46,47]. Sca are also present on the antennae and wings of some insects [48,49,50]. The majority of Sca in cockroaches (*P. americana*) are distributed on the inner side of the cerci [19]. While in crickets, Sca are generally distributed around Sf or peg sensilla. In some crickets (*Acheta domesticus*), the number of Sca is related to the diameter of the hair sockets of filiform or sensilla rod-like and the length of the sensory hairs [51,52]. In some crickets (*Acheta domesticus*), Sca play an auxiliary strengthening role in the perception process of the two types of sensilla mentioned above. Observations revealed no significant gender or interspecies differences in Sca. The proximity to Sc could indicate a synergistic role in stimulus detection.

In the sensory system of insects, CP are a special type of structure that, despite their relatively simple morphology, play a crucial role in insects’ perception of the external environment and regulation of their own physiological activities. The CP of the *H. patellifera* has a clear central hole, while the CP of the *S. maculata* has a folded structure in addition to the central hole, suggesting the complexity and specificity of CP function. Some scholars speculate that CP may be channels formed by depressions in the epidermis, primarily serving functions such as gas exchange or heat dissipation [52]. This hypothesis requires dissecting the pores and examining their internal structure using transmission electron microscopy for confirmation. Further electrophysiological studies are also needed to determine whether olfactory receptors participate in pheromone detection, humidity sensing, or other sensory modalities.

The cerci are divided into four parts based on article differences and the variety of sensilla distributed on them. The functional zoning of the cerci suggests potential sensory specializations that may correspond to ecological strategies. Part I (Proximal Basic Perception Zone), characterized by its compact structure and high density of ScII (especially in females), is postulated to be a core region for mechanoreception related to reproduction, providing essential positional and contact information during mating and oviposition. Part II (Central Comprehensive Perception Zone), with the greatest diversity and density of sensilla, likely serves as the primary hub for integrating mechanical and potential chemical cues for complex behaviors. Both species rely on this area to process key signals for mating, which is the core function of the cerci. Part III (Distal Fine Perception Transition Zone) serves as a transition zone between Part II and Part IV, with its morphology and sensilla distribution exhibiting significant “gradient changes”. Unlike Part II, “Full Sensilla Type Coverage,” Part III presents selective retention and reduction of sensilla types and density. In part III, ScII disappeared or became extremely rare, while ScI density decreased slightly but remained evenly distributed and dominant. This part has a reduced number of Sf and a very small amount of Sca/CP. Part IV (Terminal Specialized Perception Zone), as the terminal end of the tail, has the most simplified sensory repertoire, completely lacking Sf and ScII. Part III and Part IV exhibit a simplified sensory repertoire, likely specialized for direct physical contact with the environment or objects, such as the substrate during oviposition.

## 5. Conclusions

This study provides the first comprehensive ultrastructural description of the cereal sensilla in *H. patellifera* and *S. maculata*. The results revealed that although both species share four basic types of sensilla—sensilla filiformia (Sf), sensilla chaetica (Sc, including subtypes ScI and ScII), sensilla campaniformia (Sca), and cuticular pores (CP)—significant interspecific and sexual differences were observed in their size, quantity, and distribution. Specifically, *H. patellifera* possesses longer cerci with distinctive clusters of short, stout ScI sensilla in the proximal–middle region, whereas *S. maculata* displays shorter cerci predominantly covered by slender ScI. These structural differences may represent adaptations to their respective microhabitats: *H. patellifera* occupies open canopy environments, while *S. maculata* inhabits cluttered understory vegetation. In terms of sexual dimorphism, females of both species exhibited a greater number of cercal articles and longer cerci. Female-biased size dimorphism in ScI/ScII may reflect selection pressure for enhanced mechanoreception during oviposition site selection. Conversely, the higher density of Sf sensilla in males, which likely functions to detect subtle female movements or airflow changes, provides critical early warning signals.

Based on the combination and distribution gradient of sensilla, we further proposed a functional zoning model for the cerci, dividing them into four consecutive regions from base to tip: the proximal basic perception zone, central comprehensive perception zone, distal fine perception transition zone, and terminal specialized perception zone. This zonal organization reflects a functional gradient in mechanoreception and potential chemoreception, likely supporting key reproductive behaviors such as mate localization, copulatory coordination, and oviposition substrate assessment. This study not only clarifies the ultrastructural characteristics and distribution patterns of cercal sensilla in two mantis species with distinct ecological niches, but also lays a solid morphological foundation for subsequent electrophysiological and behavioral studies to verify the functional roles of these sensilla in key life activities, especially reproductive behaviors (e.g., mating, oviposition), as well as to advance research on mantis sensory ecology and phylogeny.

## Figures and Tables

**Table 1:** Morphological characteristics of cercal sensilla in male and female mantises.

Type of Sensilla	Species		Diameter (μm)	Length (μm)	Outer Wall of Sensillar Hair
	Species	Sex			
Sensilla filiformia	*H. patellifera*	male	18.1 ± 1.7 aA	113.2 ± 50.3 aA	Shallow grooved
		female	23.0 ± 2.0 aA	125.6 ± 49.3 aA	Shallow grooved
	*S. maculata*	male	20.2 ± 2.8 aA	155.2 ± 75.3 aA	Shallow grooved
		female	22.2 ± 2.2 aA	148.2 ± 66.0 aA	Shallow grooved
Sensilla chaetica I	*H. patellifera*	male	15.2 ± 2.4 aA	62.9 ± 20.4 bB	Deeply grooved
		female	17.8 ± 4.1 aA	78.2 ± 32.6 aB	Deeply grooved
	*S. maculata*	male	16.8 ± 1.2 aA	94.4 ± 24.2 bA	Deeply grooved
		female	17.3 ± 3.1 aA	116.0 ± 22.9 aA	Deeply grooved
Sensilla chaetica II	*H. patellifera*	male	21.5 ± 3.0 aA	274.0 ± 66.6 bA	Deeply grooved
		female	24.5 ± 3.4 aA	477.2 ± 76.0 aA	Deeply grooved
	*S. maculata*	male	17.0 ± 2.2 bB	220.3 ± 12.7 bB	Deeply grooved
		female	20.0 ± 3.3 aB	250.5 ± 29.4 aB	Deeply grooved
Sensilla campaniformia	*H. patellifera*	male	7.6 ± 1.0 aA	—	—
		female	7.6 ± 1.2 aA	—	—
	*S. maculata*	male	8.1 ± 0.7 aA	—	—
		female	7.4 ± 1.0 aA	—	—

Note: 1. Different lowercase letters indicate that there was a significant difference between male and female within the same species (*p* < 0.05), and different uppercase letters indicate that there was a significant difference between the same sex (*p* < 0.05). 2. The data format is ‘mean ± standard deviation’, and the sample size was *n* = 5. 3. “—” indicates not detected due to equal heights between Sensilla campaniformia and epidermis.

## Data Availability

The original contributions presented in the study are included in the article. Further inquiries can be directed to the corresponding author.
